# Assessing and treating complex mental health needs among homeless youth in a shelter-based clinic

**DOI:** 10.1186/s12913-020-4953-9

**Published:** 2020-02-11

**Authors:** Dominika A. Winiarski, Anne K. Rufa, Dawn T. Bounds, Angela C. Glover, Kristin A. Hill, Niranjan S. Karnik

**Affiliations:** 1 Department of Psychiatry and Behavioral Sciences, Section of Population Behavioral Health, Rush University Medical Center, 1645 W. Jackson Blvd., Suite 302, Chicago, IL 60612 USA; 2 College of Nursing, Department of Community, Systems, & Mental Health Nursing, Rush University Medical Center, Chicago, IL USA

**Keywords:** Homelessness, Transition-age youth, Community mental health, Shelter-based

## Abstract

**Background:**

Rates of homelessness have been increasing in recent years, thereby necessitating a more direct approach to treating this complex social problem. Homeless youth have disproportionately high rates of untreated mental health problems and are therefore particularly vulnerable to the effects of homelessness during the transition period from adolescence to adulthood.

**Methods:**

The study team developed a shelter-based clinic and collected clinical measures on youth who attended this clinic from October 2016 through June 2018.

**Results:**

Youth attended an average number of three sessions, but there was a significant drop in follow-up after the first (intake) appointment. Depression, anger, and adjustment disorder emerged as the most common presenting mental health concerns identified by clinicians in the intake appointment, and trauma was identified as a significant complaint for those youth who returned for a second session.

**Conclusion:**

Mental health care is needed in this population, but future studies should explore alternative approaches to retaining homeless youth in treatment and in designing targeted trauma-informed interventions.

## Background

From 2016 to 2017, the rates of homelessness in the United States increased for the first time in seven years [1]. On a single night in 2017, an estimated 553,742 people experienced homelessness (i.e., living on the streets, in a shelter, or in some other alternative living situation) in the United States. Approximately 40,799 of those experiencing homelessness were unaccompanied homeless youth under the age of 25, who are also 55% more likely to be unsheltered than their adult counterparts [1], and are at especially high risk for experiencing trauma and victimization [2]. These youth are therefore in greatest need of mental health services, but being part of a marginalized and underserved population often makes it much more difficult to advocate for and obtain these essential behavioral health services [3, 4]. It is therefore imperative to focus on the development of strategies for the dissemination of empirically supported care that take into account the multitude of complicating social factors impeding mental health treatment in this population.

Rates of traumatic experiences among homeless youth are high, oftentimes across both childhood and adolescence and as a consequence of being unstably housed/homeless. For example, in a large study of homeless youth from several major cities in the United States, 57% of the 146-sampled youth experienced a traumatic event and 24% met Diagnostic and Statistical Manual of Mental Disorders-IV criteria for posttraumatic stress disorder [5]. Similarly, trauma was identified as the most common risk factor for psychopathology among thirty-five homeless youth between the ages of 14–25 [6], with as many as 77% of youth reportedly experiencing physical abuse, sexual abuse, or both [7].

Previous work has shown that 89% of homeless 16–19 year-old youth met criteria for one or more mental health disorders, compared with 30% for the national population within that same age range [8]. Similarly, a representative sample of homeless youth across multiple US cities found that, in each city, more than 80% of homeless youth met criteria for at least one psychiatric diagnosis. In Chicago in particular, 62.5% of youth met diagnostic criteria for any mood disorder and 48% met criteria for a substance-related disorder. Moreover, 62.5% of youth reported suicidality [9]. In sum, multiple lines of research suggest that exposure to early life adversity correlates to higher risk for the development of future psychopathology [10–12].

For the purposes of this paper, youth are defined as those in the transition period from adolescence to young adulthood, roughly ages 16 through 25 [13]. Without adequate psychological support, an estimated 50% of transition-age homeless youth continue experiencing housing instability and/or homelessness into adulthood [14]. Mental wellness is critical for stable employment and the formation of reliable and safe interpersonal connections; thus, mental health problems can play a direct role in perpetuating the cycle of homelessness. It is therefore essential to prioritize developing mental health programs for vulnerable youth before their problems are exacerbated. One complicating factor in establishing mental health care in this population is provider mistrust. Although homeless youth present with numerous mental health and social needs, they are also less likely to seek out support from providers due to low perceptions of trust of the mental healthcare field and a fear of judgement from providers [15, 16]. Likewise, previous research also suggests that, particularly for African American men, past experiences with public service agencies more broadly (i.e., the child welfare system) can undermine their trust in service providers, including those delivering mental healthcare services [17]. Others have similarly found that negative experiences in one aspect of the healthcare system can adversely impact utilization and perception of other social and health services in the future [18]. Because being part of a marginalized and underserved population often makes it much more difficult to advocate for and obtain essential healthcare and behavioral health services [3, 4], it is not unlikely that homeless youth have frequently encountered frustrations and injustices in the healthcare system that may make them leery of interacting with this system in the future. While this is a multifaceted issue well beyond the scope of the present paper, it is worth highlighting because the development of any mental health program in this population needs to take into account how important this factor may be in treatment initiation and retention.

Previous studies have identified several different avenues by which homeless youth can access treatment for mental health concerns including emergency departments, crisis centers, community clinics, shelters, and drop-in centers [16, 19, 20]. Street outreach efforts have also been successful in engaging homeless youth in care [19, 21]. Youth shelters and drop-in centers (i.e., service agencies at which youth can receive immediate services such as food, showers, transportation fare, laundry, and case management) seem to be viewed more favorably and utilized at higher rates in this population compared to other means. DeRosa and colleagues found that in a sample of 296 homeless youth, 78% had used a drop-in center and 40% had used a youth shelter since arriving in their current city [22]. Part of the reason why mental health services are more likely to be utilized through these venues is that both may have staff trained to provide crisis services. This specialized training is valuable as homeless youth note a desire for care from providers who understand their unique life circumstances [19, 21–23].

In addition to providing “on-the-spot” services, staff at drop-in centers and shelters also serve as liaisons to the mental healthcare system by providing referrals to outside providers [16, 19] and helping youth identify strategies for obtaining insurance, if needed. While targeted interventions for homeless youth do exist, they tend to focus heavily on substance use [24–26] and sexual health, such as prevention of Human Immunodeficiency Virus and sexually transmitted infections [27, 28]. In Slesnick’s review of targeted services and interventions for homeless youth aimed at addressing these problems, it is noted that while many interventions describe a general rationale for implementation, there is limited empirical basis for the interventions most frequently utilized in this population [16]. Despite these efforts to target substance use, many youth do not express interest in this kind of treatment, and report using substances as a way to cope with the stressful circumstances of homelessness and their mental health [21, 23].

Despite the existence of treatment options available to homeless youth, research suggests that access to and use of these resources is low in this population. One study found that of 100 homeless youth who met criteria for emotional distress as measured by the Brief Symptom Inventory, only 32% used a mental health service [20]. Similarly, in a study done with 94 homeless and low-income-housed youth, Buckner and Bassuk found that 32% of the sample met criteria for a mental disorder accompanied by impairment in functioning, but only 20–35% of those had received mental health services [29]. DeRosa and colleagues found that of 296 homeless youth surveyed, only 9% had specifically sought out and used psychological services since arriving in their current city [22]. Various barriers to accessing mental health care exist, including financial, logistical, and interpersonal obstacles [15, 23, 30]. Homeless youth may be uninsured and unaware of the kinds of insurance services available to them. Moreover, homeless youth may struggle with healthcare literacy and may be unsure of how to successfully navigate the healthcare system. For example, in a study involving 688 homeless adolescents, 57% of youth who perceived a need for mental health services but did not seek them out reported that they did not know where to go or how to initiate engagement in services [20].

Even if youth are able to connect with a service, transportation to the site might be unreliable [15]. Further, homeless youth specifically worry that providers will not believe them or that they may not receive the best care with inadequate insurance [15, 23, 31]. It has also been suggested that youth in this population do not perceive a need for help regarding their mental health [23], or that they may prioritize other health needs, such as physical and sexual health. In a 2012 survey of 249 homeless youth, Tyler and colleagues found that 52.2% of the sample reported being tested for sexually transmitted infections multiple times a year while 57.4% reported never having accessed counseling services [32]. Youth also may prioritize pressing concerns such as searching for employment or housing, food and clothing, or places to wash and use the restroom over seeking mental healthcare. These competing priorities pose significant barriers to accessing mental health treatment [33].

In an effort to address the numerous barriers outlined above, the shelter-based clinic described in this paper was staffed weekly by psychologists providing mental health care to youth on-site. Youth were not required to have insurance and received all services free of charge. The current study presents mental health data (i.e., diagnostic, treatment utilization) from youth seeking mental-health services through this clinic. Suggestions for further expansion of mental healthcare access for this underserved population based on the preliminary clinical data presented here are outlined as well.

## Methods

### Setting and participants

The primary aims of this study were to (1) describe the development of a shelter-based clinical model for homeless youth, (2) to evaluate service utilization in this population, and (3) to illustrate the diverse mental health needs that youth present with for care. The study took place in a homeless shelter system serving young adults in a large Midwestern city. While definitions of homelessness vary considerably across federal and social service organizations [34], the authors defined homelessness in the present study as “lacking a fixed, regular, and adequate nighttime residence and residing in the shelter for most nights of the week.” This definition was intentionally broad to include youth served by each program of the organization running the shelter: youth in short-term housing programs, youth in the long-term (2-year) housing program, as well as youth participating in the street outreach services. Youth connected with shelter sites are assigned a case manager who provides a variety of services and support, including: ensuring they are able to attend school or enroll in programs to attain their General Educational Development certificate (GED) or high school equivalency; attaining a job; budgeting and saving money; finding and securing housing; and applying for health insurance and other relevant services (e.g., Social Security Disability). All youth connected with the homeless shelter system were eligible for free mental health services and thus for participation in this study. Youth under the age of 16 were eligible for a brief mental health assessment only, while all other youth were eligible for an initial assessment and ongoing therapy. Under the Illinois Emancipation of Mature Minors Act (750 ILCS 30), a 16-year-old minor is mature enough to consent for certain physical and mental health services without a parent. This statute allowed us to provide minors with a mental health assessment and give treatment recommendations to the youth and their case management team, but because youth under age 16 are generally taken into emergency foster care by the Department of Child and Family Services in Illinois, we could not follow them for outpatient care in our clinic. The clinic model described in this study was established in response to research conducted among a specific subsample of homeless youth [9, 14].

The clinic was initially developed in partnership between the senior author (NSK) and the shelter system beginning in 2010. Initially, the clinic was conducting neuropsychological testing as part of a broader epidemiological research study. In 2014, the clinic was advanced with support from Rush University to allow psychiatric services on an as-needed basis and psychotherapy services provided by one postdoctoral fellow in psychology. Both parties signed a Memorandum of Understanding (MOU) detailing expectations of the partnership in 2014. Services are provided on-site at the shelter in a private office at no cost. The only costs associated with the clinic are those covering staff effort, which is supported by the Rush University Medical Center Department of Psychiatry and Behavioral Sciences, as part of their general agreement to support the senior author’s (NSK) research endeavors in the community. Services are provided on-site at the shelter in a private office at no cost because the shelter network is not equipped to bill or serve as a primary health care provider.

Participants were either self-referred or referred by their case managers to mental health services, which were provided by five psychology postdoctoral fellows from a large academic medical center, between October 2016 through June 2018. Although services were initiated in 2014, procedures for routinely collecting the data reported herein were formalized at the beginning of the 2016 academic year. The clinic was staffed 2–3 times per week for approximately 4 hours per time slot, with slots scheduled during both late morning and evening hours to accommodate variability in the young adults’ schedules. The majority of the participants were residents of a shelter site providing both interim (12 months) and transitional (24 months) housing services. On rare occasions, some of the young adults traveled to this site from other sites within the shelter system or were connected through other outreach programs within the organization (i.e., if their case worker was requesting the provider’s input about acutely emerging psychosocial stressors or mental health needs). Although the shelter system provides mobile health services for street homeless youth, these youth are generally not referred for mental health care in our clinic as initial engagement of youth is focused on the youth living in the shelter sites described above. In addition, our limited staffing is a practical limitation that prevents us from participating in these street outreach efforts at this time. For those youth who did not reside at the sites hosting the clinic, transportation was either coordinated by the case manager or youth used public transportation with passes or costs covered by the shelter system. Beginning in February 2018, services were also provided on-site at a shelter providing housing to pregnant and parenting young women. This particular clinic was staffed once each week for approximately 2 hours. Typically, young children were present for treatment sessions. On occasion, staff at the pregnant and parenting shelter site were available to care for children during sessions. Youth were not incentivized for completing mental health intakes or for attending subsequent therapy sessions. Participation in this clinic did not substitute for other mental health or social service program utilization; youth had the option to pursue treatment within this clinic or obtain services through other organizations. Case management staff in the shelter network assisted youth with navigating the process of obtaining these services.

Clinical assessment measures were collected as part of standard clinical care only from psychologists providing therapy services; however, youth also had access to free psychiatric services beginning in 2014.

### Measures

#### Demographics

Study and clinical staff developed a comprehensive demographics form for youth to complete during their first mental health session. Information collected included: age, gender, sexual orientation, highest degree earned, race/ethnicity, employment/educational status, history of homelessness, and history of head injuries and/or loss (es) of consciousness.

#### Post-encounter form

These clinician rating forms capture service-related variables and were completed following each session. Clinicians rated both severity of mental health presentation and improvement in mental health status using the Clinical Global Impression Scale (CGI) [35]. The CGI is a widely used instrument in psychiatric research and clinical settings as it is brief, easy to use, and transdiagnostic. Studies of its use suggest it has a moderate-to-strong correlation with both clinician-administered and self-report measures of social anxiety and depression [36]. Additionally, it is able to meaningfully differentiate between responders and nonresponders in clinical trials of depression [37] and antipsychotic medications [38]. Both severity and improvement measurements use a 7-point Likert scale ranging from *not at all ill* to *among the most extremely ill* (severity) and from *very much improved* to *very much worse* (improvement). Improvement is rated as not applicable during the first session. Additionally, clinicians identified a primary complaint and a primary intervention used, as well as the time spent on that intervention. A secondary complaint, secondary intervention, and time spent on this intervention were rated, if applicable. Clinicians were provided with a list of possible presenting complaints based on previous literature assessing mental health needs in this population. If a clinician identified that the presenting concerns did not reliably fit into one of the pre-existing categories, (s) he had the option to list “other” and then list the client’s self-reported complaint. On this form, clinicians also had the option to select from a variety of treatment modalities, such as CBT, Intake Evaluation, Psychoeducation, Motivational Interviewing, or Supportive Therapy.

### Data analysis

Descriptive statistics were run using SPSS 22 Premium to analyze demographics features of the sample, presenting complaints and interventions used, as well as to characterize participants’ treatment utilization patterns.

## Results

Clinical data were collected on homeless youth (*N* = 77) who attended the shelter-based clinic described above from October 2016 through June 2018. Average age of participants was 19.1 (SD = 1.13, range = 14–21). Most of those who presented to the clinic were not currently enrolled in school (58.8%) and were unemployed (67.2%). Additional demographic characteristics are reported in Table 1. Demographic forms were not completed by 9 youth. Completion of this measure was optional, and did not influence whether or not youth could receive treatment in the clinic.

**Table 1:** Demographics collected at intake of youth who received mental health services

	N (%)
Gender	
Female	41 (60.3%)
Male	26 (38.2)
MTF Transgender	1 (1.5%)
Sexual Orientation	
Straight/Heterosexual	48 (70.6%)
Gay or Lesbian	9 (13.2%)
Bisexual	9 (13.2%)
Don’t Know	1 (1.5%)
Other	1 (1.5%)
Race	
Black or African American	43 (55.8%)
White/Non-Hispanic	3 (3.9%)
Mixed/No Primary Affiliation	13 (16.9%)
Other	7 (9.1%)
Don’t Know	1 (1.3%)
Refused/Don’t Know	10 (13.0%)
Ethnicity	
Hispanic/Latino/Spanish Origin	17 (22.1%)
Highest Level of Education	
Less than High School	27 (40.3%)
Regular High School Diploma	23 (34.3%)
GED or alternative credential	7 (10.4%)
Vocational/training school after High School	1 (1.5%)
Some College or Associate Degree	9 (13.4%)

There was a significant range in age of first episode of homelessness (range = 7–21), and most participants reported being homeless one or two times (56.7%). In addition, 30.3% (*N* = 20) of youth reported that they had sustained a head injury, and 12.5% (*N* = 8) reportedly lost consciousness as a result of their head injury.

A plurality of youth only attended the first intake session (49.4%). Second session follow-up appointments sharply declined (13%), and gradually declined thereafter. The average number of sessions attended was three (SD = 4.1, median = 2), but there was a significant range (1–25). Because the majority of youth only attended an average of three sessions, clinician data on presenting complaints and overall client mental health functioning data are only presented for these three sessions. On the CGI, most youth (83.8%) were rated as moderately-to-severely ill at intake. In the intake sessions, the most common clinician-identified presenting complaints were Depression (22.1%), Adjustment Disorder (16.9%), and Anger (15.6%). Because most youth only completed the first session, the most commonly selected clinician intervention was the intake assessment (83.1%) at this time point. For later sessions, supportive therapy and CBT were the most commonly selected treatment modalities. (CBT: ≥26.7% in sessions 2 and 3; Supportive therapy ≥25.6% in sessions 2 and 3). Of the youth who returned for a second session, the most common clinician-identified presenting complaints were Depression (23.1%) and Trauma (12.8%). This trend continued such that both Depression (13.3%) and Trauma (16.7%) were the most common complaints for youth who returned for a third session. Table 2 lists the clinician-reported presenting complaints from sessions 1 through 3. The most common “other” presenting complaints were Bipolar Disorder and general social issues (e.g., lack of support in pregnancy, difficulty obtaining employment, homelessness). Clinically significant changes on the CGI were not observed for youth who attended the mean number of therapy sessions (3), *t* (6) = 1.549, *p* = 0.172, *d* = 0.43.

**Table 2:** Primary presenting complaints for sessions 1–3

	N (%)
Session 1	
Adjustment Disorder	13 (16.9%)
Anger	12 (15.6%)
Anxiety	4 (5.2%)
Depression	17 (22.1%)
Family Problem	1 (1.3%)
Relational Problem	3 (3.9%)
Trauma Reaction	5 (6.5%)
Other*	14 (18.2%)
Multiple Complaints Listed	5 (6.5%)
Missing/Not Listed	3 (3.9%)
Session 2	
Adjustment Disorder	2 (5.1%)
Anger	3 (7.7%)
Anxiety	3 (7.7%)
Depression	9 (23.1%)
Family Problem	3 (7.7%)
Relational Problem	2 (5.1%)
Substance Use	2 (5.1%)
Trauma Reaction	5 (12.8%)
Other*	9 (23.1%)
Multiple Primary Complaints	1 (2.6%)
Session 3	
Adjustment Disorder	3 (10.0%)
Anger	2 (6.7%)
Anxiety	2 (6.7%)
Depression	4 (5.2%)
Family Problem	1 (1.3%)
Impulse Control	1 (1.3%)
Relational Problem	3 (3.9%)
Substance Use	1 (1.3%)
Trauma Reaction	5 (6.5%)
Other*	5 (6.5%)
Multiple Primary Complaints	2 (2.6%)
Missing/Not Listed	1 (1.3%)

*Note: See in-text description of presenting complaints listed as “other”

## Discussion

Overall, descriptive clinical data from the shelter-based clinic described in this paper illustrate the need to identify alternative strategies for retaining homeless youth in mental health treatment. Although preliminary data suggest that some youth are motivated to return for follow-up care, there is nevertheless a sharp decline in the number of youth who attend more than one (i.e., intake) session. Consistent with previous literature [8, 15], results from this sample of youth also suggest that mental health concerns are high, with depression, anger, and adjustment disorder emerging as the most common presenting mental health concerns identified by clinicians in the intake appointment. The development of future mental health treatment programs needs to account for potential barriers to care while being sensitive to the unique mental health needs that youth disclose to providers during the intake process. Of note, trauma was not identified as a primary concern until the second visit which suggests that trauma may often be related to homeless young adults’ presenting complaints but may take additional time to uncover. Youth may also not feel comfortable disclosing traumatic experiences to a new provider in the intake session. Of note, the lack of clinically and statistically significant findings in reductions on the Clinical Global Impression Scale (CGI) are not surprising; since the average number of sessions was 3 and the first session was primarily an intake session, we would not expect to see clinically meaningful symptom reduction in such a few number of sessions. Future directions may evaluate whether there are other important changes that occur outside of symptom reduction, such as improved trust in providers or increased willingness to participate in future mental health treatment.

When developing future interventions for this population, it is important to keep in mind the treatment goals of the youth and/or the case management team. Anecdotally, many of the case managers at the Night Ministry identified substance use as a primary treatment target they would like the clinicians to address. However, as the data presented in this paper show, very few youth identified substance use as a presenting concern (and none did so in the first session), thereby illustrating a clear disconnect between the assumed needs and treatment targets of the youth and of the support staff working in the shelters. If additional substance use interventions are designed to specifically target homeless youth, it would be helpful to address these specific concerns from a motivational interviewing framework to increase buy-in and acceptability.

Similarly, while anger was identified as a common presenting complaint in the intake session, very few youth followed up for anger management in subsequent sessions. It could be that youth with severe anger problems were dismissed from the program for behavioral/safety concerns, or that they attended the intake session at the request of their case manager, but had no interest in engaging in services beyond the first session. One limitation of this study was the providers’ inability to follow-up with youth post-discharge from the program to identify ongoing mental health needs, but properly addressing this limitation requires that several important issues be considered. Balancing the needs of shelters (i.e. managing disruptive behavior, aggression and conflicts amongst residents) with the rights of young adults (i.e. autonomy, shared decision making, and prioritizing basic survival needs) can be quite challenging. On one hand, shelter staff have an obligation to maintain a safe, secure space for all residents. However, on the other hand, most of the residents are legally adults who have the right to refuse shelter staff’s mandates to seek mental health treatment and take prescribed medication. This disconnect impacts treatment referrals as well as commitment to follow up. This conflict in priorities also places mental health providers in the middle of complex ethical issues. Mental health providers have an ethical obligation to offer informed consent that is not connected to keeping housing or other basic needs despite shelter rules that might require adherence to treatment as a condition to remain in the shelter. Navigating the space between shelter staff and residents’ needs creates an opportunity for mediation, advocacy, and psychoeducation.

While not a primary focus of the paper and beyond the scope of services available in the clinic at this time, it is important to recognize the high frequency of self-reported head injuries by youth in this sample. The associations between head injury and both cognitive and emotional sequelae have been long well-established in the medical literature, and this relationship is dependent both on severity and frequency of the injuries [39, 40], and may be underlying some of the problems that drove youth into homelessness and are further perpetuating the cycle of homelessness. Thus, in addition to traditional outpatient therapy, it is important to find ways to increase access to neuropsychological testing and related services (Individualized Education Plan/504 Plan support, etc.). Likewise, future work should also include an assessment of psychiatric services offered to homeless youth in this and similar clinical settings to evaluate whether youth engage with psychiatric services differently than with psychological resources.

Of the twelve youth who were seen for more than 6 sessions, five were in the long-term (24-month) housing program. Two of the youth were diagnosed with severe mental illness, and required regular mental health consultations as part of their case management care plan. It is possible that the youth in the 24-month program experienced less pressure to find housing and had stable employment/schooling (a requirement to remain in the long-term program), and therefore had more stability to benefit from ongoing mental health treatment. In contrast, youth in the short-term housing programs were often working to find long-term housing, interviewing for/seeking out jobs, and some were attending school. Scheduling therapy around these competing demands can be a logistical challenge. Challenges with mental health may prevent youth from being successful in their academic and career endeavors.

Based on the findings from this sample, it appears that simply addressing a logistical barrier (i.e., convenience/clinic location) is not enough to significantly improve patient retention in mental health services. An important limitation of the present study is that, to date, scheduling and communicating with youth between sessions has been largely facilitated through case managers. In the future, clinicians in our program may explore means of more direct contact with youth if they are interested in providing contact information for follow-up after the first session. Text message-based appointment reminders and communication may also facilitate follow-up [41, 42]. Related to this point, it is important to evaluate specific reasons why youth did not follow-up for services in the clinic. Before targeted approaches are developed, it is important to know what services youth want, what they are willing to engage with, how logistical barriers can be more effectively overcome, and how youth can remain engaged beyond the first session. Future research should explore how targeted approaches can be integrated into other programs such as education attainment and/or job placement.

Similarly, a future research direction is to complete focus groups or individual interviews with homeless youth to gather additional qualitative information on service utilization. While previous studies have established that there are high rates of mental health problems in this population, our work suggests that retention in services is an equally important problem. Youth should play an active role in evaluating ideas for future treatment development, as previous work has demonstrated the need to better understand the specific perceptions of and attitudes toward mental health and treatment options in order to increase quality mental health care for higher-risk populations.

One proposed strategy for reaching youth more consistently has been to harness technology to disseminate empirically-based mental health tools to youth. For example, previous studies have found high acceptability of a cell-phone based intervention in which youth were provided with phones and data plans for 1 month [43]. The phones came preloaded with mental health apps designed to address mood regulation, sleep, and teach basic cognitive-behavioral principles. Youth were also given the option to schedule three, 30-min phone therapy sessions with a doctoral-level therapist. Youth reported high levels of satisfaction with the study (i.e., 70% reported being moderately-to-extremely satisfied with the study, and 90% would recommend participation in the study to others), and most utilized all three 30-min phone therapy sessions. In a fully-automated follow-up to this study, youth also reported being very willing to engage with the features of the intervention. Thus, these data collectively illustrate that technology can provide a fruitful avenue by which mental health services can be provided to youth, particularly those who are unstably housed and have the greatest number of barriers to formal care. Future research should more closely evaluate whether these fully automated programs are equally efficacious to clinical standards-of-care (e.g., Cognitive Behavioral Therapy).

Given the limited time homeless youth engage in mental health care, interventions tend to be brief and supportive in nature. Although a specific intervention or approach might be indicated for the presenting problem or diagnosis, without a commitment to ongoing treatment, mental health providers are faced with choosing an eclectic approach instead of what is indicated. Some might argue that a non-empirically based intervention is better than no treatment at all. Utilization of a transdiagnostic model may offer some guidance in these situations [44]. Models like the Common Elements Treatment Approach (CETA) teach patients eight key cognitive behavioral tools that have been found to yield positive clinical outcomes. This approach has been successful among individuals experiencing trauma-related disorders [45, 46] and in low resource countries [47], thereby making this approach particularly useful for homeless youth.

An alternative view is to conceptualize the first session as the primary intervention point, and to therefore focus clinical research efforts on designing brief, targeted, and single time-point interventions for this population. The greatest attention should be paid to developing clinical tools that target depression and trauma, as these are among the most-commonly reported mental health concerns both in this sample and in previously-studied representative samples of homeless youth across the United States [5, 6, 9]. Relatedly, previous qualitative research with youth residing in supportive housing has demonstrated the importance of designing housing programs that directly address needs of youth who are working on building their self-efficacy and independence, and who sometimes report feeling like they receive mixed and inconsistent messaging regarding the need to develop greater autonomy while simultaneously being expected to abide by very strict rules (characteristic of being treated like a child) [48]. If we extrapolate these findings on attitudes on supportive housing to the findings presented here, it would appear that youth may prioritize services that focus on supporting their independence and building self-efficacy that they have not had in the past over targeting specific mental health diagnoses or conditions. Again, future qualitative research with youth in the shelter may help us better identify factors that motivate ongoing mental health treatment in this population.

Likewise, given the high levels of mistrust toward adults and the mental healthcare system [16], clinicians should consider finding appropriate ways to engage youth in serving as bridges to formal mental health care, such as through the creation of youth mental health ambassador programs. Clinicians working with homeless youth and other historically-marginalized populations may also consider explicitly measuring cultural mistrust, such as through tools like the Medical Mistrust Index (MMI [18];), to gauge how much time in therapy needs to be spent on directly addressing this issue as a way to potentially increase compliance in follow-up care. These programs could be loosely based on the Friendship Bench model [49–51], which has shown promising results in developing countries where access to empirically-based care is limited, or on the CETA model described above, which has been used by lay mental health workers in developing countries [47]. Briefly, the Friendship Bench program trained laypersons in the delivery of an empirically-supported mental health treatment program for common mental health disorders (e.g., depression, anxiety, etc.). Laypersons were formally supervised by licensed mental health professionals. Youth “ambassadors” may have greater social clout than licensed professionals, particularly in cases where youth have had adverse experiences with the social justice system (including mental healthcare workers, juvenile justice, etc.), and could therefore serve as liaisons in the reintegration of underserved youth into the mental healthcare system [52]. To the authors’ knowledge, such programs have yet to be developed and evaluated in the United States, but data from low- and middle-income countries suggest that utilizing laypersons helps to build trust in, and expand access to, the mental healthcare system. In any case, greater priority needs to be taken to directly measure and assess these needs among young adults specifically [53], as opposed to extrapolating findings from the adult and adolescent literatures for this transitional age group. Similarly, as has been identified in previous studies, rather than simply focusing on determinants of program engagement, future iterations of clinical service models for homeless youth need to focus on identifying appropriate ways to communicate with youth [54]], particularly around issues of initiating and sustaining mental health treatment.

## Conclusions

Overall, the results from this paper suggest that youth make initial contact with providers in a shelter-based mental health clinic, but that more information is needed to understand strategies for retaining youth in care beyond the first session. Likewise, results from this shelter-based clinic sample suggest that more work is needed to provide short-term and targeted trauma-based interventions in this population. Relatedly, clinicians should find ways to harness technology to develop single time-point interventions that can effectively create bridges to long-term care for homeless youth with significant mental health needs.

## Data Availability

The datasets generated and/or analyzed during the current study are not publicly available due to the sensitive nature of mental health data that were collected, but deidentified data are available from the corresponding author on reasonable request.

## Abbreviations

CETA: common elements treatment approach
CGI: clinical global impressions scale
GED: general educational development
